# Thermal Proteome Profiling Reveals Meltome Upon NLRP3 Inflammasome Activation

**DOI:** 10.1016/j.mcpro.2025.100972

**Published:** 2025-04-16

**Authors:** Chen Yang, Ling Wang, Yuchen Liu, Yuehui Zhang, Chaozhi Jin, Jiale Cheng, Limin Shang, Longlong Fang, Shanshan Wu, Chuan Chen, Jian Wang

**Affiliations:** 1 College of Life Sciences, Hebei University, Baoding, China; 2 State Key Laboratory of Medical Proteomics, Beijing Proteome Research Center, National Center for Protein Sciences (Beijing), Beijing Institute of Lifeomics, Beijing, China; 3 School of Basic Medical Sciences, Anhui Medical University, Hefei, China

**Keywords:** thermal proteome profiling, meltome, NLRP3, inflammasome, FAM120A

## Abstract

NOD-like receptor (NLR) family pyrin domain containing 3 (NLRP3) involves in inflammasome complex assembly and innate immunity. Activation of the NLRP3 inflammasome induces conformational alterations in protein complexes, influencing their interactions with other molecules, which in turn affects protein thermal stability. To investigate the proteome-wide thermal stability alterations induced by NLRP3 inflammasome activation, we conducted a comprehensive analysis of meltome dynamics using thermal proteome profiling. Our analysis identified 337 proteins exhibiting alterations in thermal stability upon NLRP3 inflammasome activation. Subsequently, we validated three proteins by the cellular thermal shift assay. Notably, our findings reveal that the majority of these proteins tend to cluster into distinct macromolecular complexes. Furthermore, we identified FAM120A as a novel NLRP3 binding partner, with its suppression enhancing caspase-1 activation and IL-1β release in response to NLRP3 agonist. Collectively, these data provide a comprehensive framework for understanding the mechanisms of NLRP3 inflammasome activation and underscore the utility of thermal proteome profiling in exploring proteome-wide thermal stability changes during signaling transduction.

## Highlights

• Thermal stability of 337 proteins changed during NLRP3 inflammasome activation.
• Integration of TPP and AP-MS to identify NLRP3 complex-associated proteins.
• FAM120A interacts with NLRP3 and ASC.
• FAM120A suppresses NLRP3 inflammasome activation and assembly.

## Graphical Abstract

**Figure:**
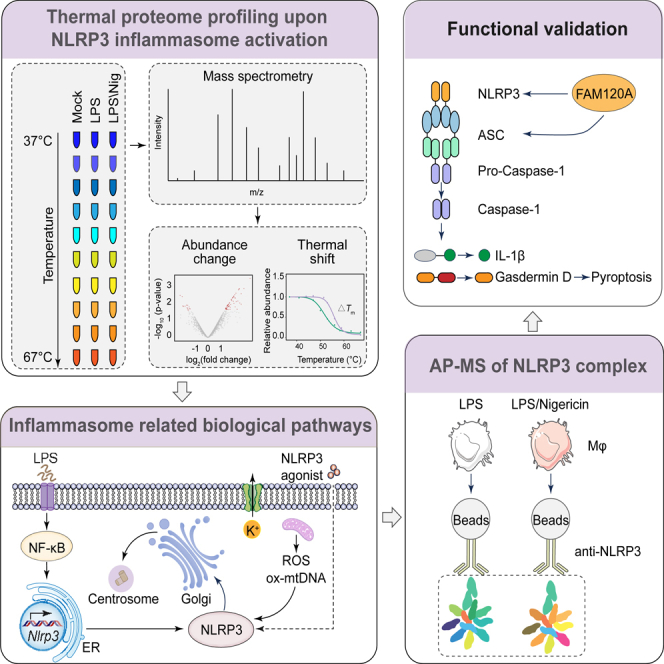

## In Brief

We used thermal proteome profiling to explore the molecular dynamics of NLRP3 inflammasome. By combining TPP with AP-MS, we identified FAM120A as a novel regulatory protein that interacts with NLRP3 and ASC, inhibiting inflammasome assembly and activation.

Infections and tissue damage triggers innate immunity and inflammation, which is mediated through the recognition of pathogen-associated molecular patterns and danger-associated molecular patterns by pattern recognition receptors (1, 2, 3). The inflammasomes are cytosolic multiprotein complexes typically consisting of pattern recognition receptors (e.g., NOD-like receptors, NLRs), adaptor protein (apoptosis-associated speck-like protein containing a CARD, ASC), and caspase (4). While multiple inflammasomes have been identified, NLRP3 inflammasome remains the most extensively characterized. Structurally, the NLRP3 protein consists of three domains: the amino-terminal pyrin domain (PYD), the nucleotide-binding NACHT domain with ATPase activity, and a carboxy-terminal leucine-rich repeat domain (LRR) (5). Inflammasome activation in macrophages requires two distinct signals. The priming signal (signal 1), typically induced by ligands binding to toll-like receptors, often utilizes bacterial lipopolysaccharide (LPS) to upregulate the expression of NLRP3, pro-IL-1β, and pro-IL-18 through activation of the transcription factor nuclear factor κB (NF-κB). The activation signal (signal 2), provided by various stimuli such as ATP, nigericin, crystalline substances, nucleic acids, and hyaluronan, initiates the assembly and activation of the NLRP3 inflammasome. The assembly of NLRP3 inflammasome facilitates the conversion of pro-caspase-1 into active caspase-1, which subsequently processes pro-IL-1β and pro-IL-18 into their mature forms (6). Mature IL-1β serves as a proinflammatory cytokine, orchestrating the recruitment of innate immune cells and modulating adaptive immune responses (7), while IL-18 promotes interferon-gamma production and enhances the cytolytic functions of natural killer and T cells (8). Activated caspase-1 also drives gasdermin D-mediated pyroptosis, releasing inflammatory mediators into the extracellular space (9).

The NLRP3 inflammasome senses a broad spectrum of pathogen-associated molecular patterns and danger-associated molecular patterns, despite lacking direct interaction with these effectors (10, 11). Instead, NLRP3 activation is thought to converge upon common cellular perturbations, including potassium or chloride efflux (12), calcium flux (13), lysosomal disruption (14, 15), mitochondrial dysfunction (16, 17), metabolic reprogramming, and trans-Golgi disassembly (18). Despite significant progress in elucidating these upstream signaling events, the pathways often intersect, and conflicting data persist (5). Notably, the dysfunction of NLRP3 inflammasome results in various diseases, including Alzheimer’s disease, gout, atherosclerosis and so on (19).

To systematically investigate proteome alterations induced by NLRP3 inflammasome activation, we employed thermal proteome profiling (TPP) to analyze meltome dynamics. TPP, combined with multiplexed quantitative mass spectrometry (MS), has been widely utilized to reveal context-specific changes in protein states, including the identification of drug targets (20, 21), metabolites binding proteins (22), and peptide-associated proteins (23), as well as large-scale assessments of protein dynamics throughout the lifespan (24). The full potential of TPP continues to be explored, including identifying differential protein complexes and functional proteome alterations (25, 26, 27, 28, 29, 30, 31, 32).

Global analyses of protein changes during NLRP3 inflammasome activation have predominantly relied on transcriptomic and proteomic studies (33, 34). Evidence indicates that numerous protein-protein interactions and conformational changes occur during NLRP3 inflammasome activation (11). These functional changes are frequently reflected in alterations to protein thermal stability (35), making TPP an invaluable tool for investigating protein state dynamics *in situ* across the proteome. By capturing these changes during NLRP3 inflammasome activation under extracellular stimulation in living cells, TPP provides a robust orthogonal approach to elucidate the molecular mechanisms underlying inflammasome function. This study leverages TPP to offer new insights into the proteomic landscape of NLRP3 inflammasome activation, advancing our understanding of its dynamic and complex molecular processes.

## Experimental Procedures

### Cell Culture and Transfection

HEK293T, immortalized bone marrow-derived macrophage (iBMDM), HeLa cells, and *Fam120a*-KO iBMDM were cultured in Dulbecco’s modified Eagle’s medium (plus 10% fetal bovine serum, supplemented with 1% penicillin–streptomycin) and maintained in a humidified 5% CO_2_ incubator at 37 °C.

Approximately five million HEK293T cells were plated per 10-cm dish and transfected with indicated constructs after 16 to 20 h. Polyethylenimine (PEI; Polysciences) was formulated to a concentration of 1 mg/ml and used for transfection of the plasmids. After incubating DNA and PEI at a 1:5 (M/V) ratio in a serum-free medium for 20 min, the transfection mix was added to the culture plate. For knockdown assay, bone marrow-derived macrophage (BMDM) cells were transfected with Lipofectamine RNAiMax (Thermo Fisher Scientific) according to the manufacturer’s instructions.

The stably transfected ASC-GFP HeLa and HEK293T cell lines and *Fam120a*-KO iBMDM cell lines were infected with lentivirus, cultured in the same medium and selected by 4 μg/ml Puromycin (MCE, HY-K1057).

### Bone Marrow Cell Isolation and Differentiation to Macrophage

All animal experiments were approved by the Institutional Animal Care and Use Committee of Beijing Institute of Lifeomics. To generate BMDMs, 6∼8-week-old C57BL/6J male mice were sacrificed. Bone marrow cells were collected in a sterile 15 ml conical centrifuge tube on ice followed by centrifugation at 1100 × rpm for 5 min. The cells were plated onto a sterile plastic Petri dish and cultured in RPMI 1640 medium supplemented with 50 ng/ml macrophage colony-stimulated factor (PeproTech, 315–02) and 10% fetal bovine serum. The cells were not rocked, and the culture medium was changed after 3 days. The cells were cultured in a humidified incubator with 5% CO_2_ at 37 °C for 7 days.

### Inflammasome Activation Assay

iBMDM cells were primed with 1,000 ng/ml lipopolysaccharide (LPS, Sigma-Aldrich, L4391) for 6 h, followed by stimulation with 10 μM nigericin for 30 min for activation. BMDM cells were primed with 400 ng/ml LPS for 6 h and then stimulated with 10 μM nigericin for 30 min for activation. We established three experimental conditions: mock-treated cells, LPS-primed cells, and LPS-primed/nigericin-stimulated cells. After stimulation, both supernatants and cell lysates were collected separately for further analysis.

### Preparation of Cell Extract for CETSA with Western Blot Readout

iBMDM cells from different treatments were harvested and washed with PBS. The cells were then resuspended in Hepes buffer (20 mM Hepes, 50 mM NaCl, 1 mM NaF, 0.5% Triton-X100, 2 mM Na_3_VO_4_, and complete protease inhibitor cocktail [Selleckchem, B14002]). The cell suspensions were lysed on ice for 20 min. The soluble fraction was separated from the cell debris by centrifugation at 100,000*g* for 25 min at 4 °C. For the cellular thermal shift assay (CETSA) melting curve experiments, cell extracts were prepared by diluting the lysates with Hepes buffer and incubating on ice for 20 min. The soluble fraction was separated from the cell debris by centrifugation at 100,000*g* for 20 min at 4 °C. The resulting supernatants were heated individually at different temperatures for 3 min in a thermal cycler (Applied Biosystems T100 Thermal Cycler, Life Technologies), followed by cooling for 3 min at room temperature. The heated cell extracts were then centrifuged at 200,000*g* for 20 min at 4 °C to separate the soluble fractions from precipitates. The supernatants were transferred to new 1.5 ml microtubes for analysis by Western blot.

After separation by SDS-PAGE, proteins were transferred to a nitrocellulose membrane, which was blocked with 5% skimmed milk. The membrane was then incubated with primary antibodies for the detection of designated proteins. The following primary antibodies were used: NLRP3 (AdipoGen, AG-20B-0014-C100), ASC (AdipoGen, AG-25B-0006-C100), β-actin (Sigma-Aldrich, A5441), Caspase-1 (p20) (AdipoGen, AG-20B-0042-C100), and IL-1β (Abcam, ab9722). The membrane was washed three times with Tris-buffered saline containing 0.05% Tween-20 and incubated with the corresponding secondary antibody for 40 min at room temperature. Chemiluminescence intensities were detected and quantified using the ChemiDoc XRS + imaging system with Image Lab software (Bio-Rad). Data from multiple runs (n ≥ 3) were plotted using OriginPro 2017 software.

### Preparation of Cell Extract for TPP

Suspension cultures of iBMDM cells were centrifuged at 340*g* for 2 min at 4 °C. The cells were then resuspended in cold PBS. After a second centrifugation step, the cells were resuspended in ice-cold PBS and centrifuged again at 340*g* for 2 min at 4 °C. The cells were then resuspended in ice-cold Hepes and lysed on ice. The samples were subjected to ultracentrifugation at 100,000*g* for 20 min at 4 °C. The protein concentration of the supernatant was determined using the bicinchoninic acid assay (Beyotime), and aliquots were used in subsequent thermal shift assays.

### Thermal Profiling Using Cell Extract

The supernatant was divided into 10 aliquots of 80 μl and transferred into 0.2-ml PCR tubes. Each aliquot was heated for 3 min at a designated temperature within the range of 37 °C to 67 °C (37, 41, 44, 47, 50, 53, 56, 59, 63, and 67 °C), followed by a 3-min incubation at room temperature. The extract was then centrifuged at 100,000*g* for 20 min at 4 °C. The supernatant was subjected to SDS-PAGE for protein separation and prepared for MS analysis.

### Sample Preparation for MS Identification

After separation on SDS-PAGE, the gels were stained with Coomassie blue. The gel lanes were cut into 1 × 1 × 1 mm cubes and subjected to in-gel digestion. To each sample tube, 500 μl of trypsin solution (10 ng/μl) was added, and the samples were incubated on ice for 30 min. They were then transferred to a 37 °C shaker at 940 rpm for 5 to 10 min. If needed, additional trypsin solution or 50 mM TEAB was added to ensure alignment with the gel surface, and the digestion continued at 37 °C for 18 h. The gel cubes were washed with 25 mM triethylammonium bicarbonate (Sigma-Aldrich, T7408) until they became transparent. Proteins were reduced with 10 mM dithiothreitol (DTT, Solarbio LIFE SCIENCES, D8220) at 56 °C for 45 min, followed by alkylation with 30 mM iodoacetamide (Sigma-Aldrich, I1149) in the dark at room temperature for 15 min. Sequencing-grade porcine trypsin (Promega) was added to the gel cubes at a final enzyme-to-substrate ratio of 1:50, and the samples were incubated at 37 °C for 16 h. Peptides were sequentially extracted using 50% acetonitrile (ACN, Sigma-Aldrich, 34851)/0.1% trifluoroacetic acid (TFA, ACROS, CAS: 76–05–1) and 80% ACN/0.1% TFA at 37 °C for 30 min. Peptide samples were then separately labeled with 10-plex tandem mass tag (TMT) (TMT10, Thermo Fisher Scientific, 90110) reagents. Each group was labeled with TMT reagents 126 to 131 (126, 127L, 127H, 128L, 128H, 129L, 129H, 130L, 130H, and 131), corresponding to a temperature gradient from 37 °C to 67 °C across the 10 samples. The labeling reaction was performed in 50 mM triethylammonium bicarbonate, pH 8.5, at 24 °C and quenched with 5% hydroxylamine. Labeled peptide extracts were combined into a single sample per experiment, acidified, desalted with C18 Sep-Pak cartridges (Waters), and then dried by vacuum centrifugation.

### Liquid Chromatography-Tandem Mass Spectrometry Analysis

Lyophilized labeled peptides were redissolved in solvent A (5 mM ammonium formate, 2% ACN, pH 10.0) and automatically loaded onto the column with flow rate of 700 μl/min for 45 min. Peptides were fractionated by basic reverse phase liquid chromatography using L-3000 HPLC System (RIGOL Technologies) with 25 cm column (C18, 5 μm, 4.6 mm × 250 mm, 300 Å) from Waters and dried by vacuum centrifugation.

Peptides were analyzed using Orbitrap Fusion Tribrid (Thermo Fisher Scientific) coupled to an EASY-nano-LC 1000 system (Thermo Fisher Scientific). Lyophilized peptides were redissolved in solvent A (solvent A, 0.1% formic acid (FA) in water; solvent B, 95% ACN/0.1% FA) and automatically loaded onto a homemade trap column (100 μm × 2 cm; particle size, 3 μm; pore size, 120 Å; SunChrom) at a flow rate of 10 μl/min. The sample was subsequently separated with a homemade analytical column at a flow rate of 600 nl/min (150 μm × 12 cm; particle size, 1.9 μm; pore size, 120 Å; SunChrom). The electrospray voltage of 2.3 kV versus the inlet of the mass spectrometer was used. Survey full-scans were acquired from 300 to 1400 m/z at a resolution of 120,000 and the maximum injection time (MIT) was set to 100 ms or an automatic gain control (AGC) target of 5e5. The 20 most intense precursors were selected for fragmentation per cycle with dynamic exclusion time of 18 s. The activation type was higher-energy collision dissociation (HCD) with a normalized collision energy of 35%. MS/MS scan were performed at a resolution 60,000, with a MIT of 118 ms or an AGC target of 5e4.

### Peptide and Protein Identification

Mascot 2.4 (Matrix Science) was used for protein identification by using a 10 parts per million mass tolerance for peptide precursors and 20 mD (HCD) mass tolerance for fragment ions. The MS data was searched against a UniProt mouse (*Mus musculus*) database (release 20200319) which includes both reviewed and unreviewed entries, totaling 86,450 protein sequences. Carbamidomethylation of cysteine residues and TMT modification of lysine residues were set as fixed modifications and methionine oxidation, and N-terminal acetylation of proteins and TMT modification of peptide N-termini were set as variable modifications; enzyme, trypsin; missed cleavages, 2; false discovery rate (FDR), <1%. The TMT reporter ion quantification analysis followed the established protocol described in Nature Protocols (2015, 10:1567–1593). Specifically, spectra with an S21 (Signal-to-Interference Measure) < 0.5 were discarded to exclude data with significant coisolation interference. Additionally, spectra with a P2T (Precursor-to-Threshold Ratio) < 4 (indicating low signal-to-noise) were filtered out to retain only high-quality precursor signals.

### TPP Analysis

The programming language R (https://www.r-project.org/) was used to analyze the raw output data from IsobarQuant (36). The datasets were processed (TPP-TR data) using a similar base workflow as described in the following: First, the output files of IsobarQuant were loaded into R and merged. The raw data were then saved in an ExpressionSet R-object. Subsequently, potential batch effects were removed using limma (37) and data were normalized using variance stabilization, vsn strategy (38). For each temperature, an independent normalization run was used in order to account for the decreasing intensity of signal sums with increasing temperature. To calculate the abundance and stability scores, we used a bootstrap algorithm; for calculating the expression scores, the results of the concordant limma analysis were modified. For all scores, two different FDRs were estimated, a local and a global FDR. The global FDR was calculated based on the score distribution (z-distribution) and thereby correlates with the effect size of the score. The local FDR, on the other hand, describes the quality and the reproducibility of the score values and takes into account the variance between replicates.

For the TPP-TR data, a limma analysis was performed on the normalized data by pairwise comparison. The resulting fold changes (logFC) were transformed into z-scores using the Cox method (39) in order to determine the expression scores for each protein. The global FDR for the expression score was calculated from the z-values using fdrtool R package taking the q-value as the output. The local FDR for the expression score was calculated by adjusting the limma *p* values for multiple testing by Benjamini and Hochberg with the p.adjust method (method = “fdr’’).

In order to calculate the abundance and stability scores, a bootstrap method was used with 500 iterations and a prerequisite of at least two measured data points for the two lowest temperatures. In the bootstrap loop, one comparison group-ratio point out of the two replicates for each temperature was chosen and used for the calculation. The abundance value was simply the average of the log2 (comparison group ratio) for the first two temperatures (37 and 41 °C). The stability value was calculated by first subtracting the average log2 (comparison group ratio) of the first temperatures from all log2 (comparison group ratios) of all temperatures and then calculating the sum of the resulting values. After repeating this 500 times, each protein yields a distribution of abundance and stability values. From these distributions, we calculated the standard deviation and the average. Together with the number of data points that were considered for bootstrapping, we could calculate a *p* value (transformation into z-scores and then assuming Student’s t-distribution) to estimate the likelihood that the distribution is different from zero (no change). The averages were transformed into the z-distribution which represent the new stability and abundance scores. The global FDR was then calculated using the fdrtool package. In order to make a call on significant hits based on these different scores, we chose a cutoff of 0.01 for the local, as well as the global FDR for each comparison and score.

Differentially expressed proteins were analyzed for Gene Ontology enrichment using the clusterProfiler package in R. Hierarchical clustering of differential proteins was performed using Perseus software. Protein interaction data were obtained from STRING (https://www.string-db.org), and interaction network maps were generated using Cytoscape software.

### Protein Thermal Stability Analysis

The melting curves were fitted with the following equation using OriginPro 2017:

ƒ(T) = $\frac{1-\text{plateau}}{1+e^{-\left(\frac{a}{T}-b\right)}}$ +plateau, where T is the temperature, a, b, and “plateau” are constants. The value of ƒ(T) at the lowest temperature Tmin was fixed to one. When the temperature is at 37 °C, the value of ƒ(T) is 1.

### Affinity Purification-Mass Spectrometry

BMDM cells were primed with 1 μg/ml LPS for 6 h, followed by stimulation with 10 μM nigericin for 30 min for activation. Two experimental conditions were established: LPS-primed cells and LPS-primed/nigericin-stimulated cells. The cells were resuspended in NETN buffer (1 M Tris-Cl pH 8.0, 0.5 M EDTA, 5 M NaCl, 0.5% NP-40, supplemented with 1 mM NaF, 0.5% Triton-X100, 2 mM Na_3_VO_4_, and a complete protease inhibitor cocktail). The cells were then lysed by sonication, and the cell lysate was centrifuged at 12,000 rpm for 10 min at 4 °C. Sixty microliters of the supernatant was mixed with 60 μl of 2 × loading buffer and heated at 100 °C for 10 min. The remaining supernatants were transferred to new tubes, and protein A/G Magnetic Beads (MedChemExpress, HY-K0202) preincubated with NLRP3 antibody (AdipoGen, AG-20B-0014-C100) for 4 h were added and incubated overnight. The magnetic beads were washed twice with 100 mM NaCl IP buffer and once with 500 mM NaCl IP buffer. The beads were resuspended in 60 μl of 100 mM NaCl IP buffer, and an equal volume of 2 × loading buffer was added. The beads were then boiled at 100 °C for 10 min. The immunoprecipitated proteins were then subjected to sample preparation for MS (as described above).

The peptides were then resuspended in 0.1% FA and analyzed using an ultraperformance LC-MS/MS platform. The LC separation was performed on an Easy nLC 1000 (Thermo Fisher Scientific) with an in-house packed capillary column (150 μm I.D. × 12 cm) with 1.9 μm C18 reverse-phase fused-silica (Michrom Bioresources). The sample was eluted with a 78 min nonlinear gradient ramped from 5 to 95% mobile phase B (phase A: 0.1% FA in water and phase B: 0.1% FA in ACN) at a 0.6 μl/min flow rate. Eluted peptides were analyzed using a Q-Exactive mass spectrometer (Thermo Fisher Scientific). The MS1 was analyzed over a mass range of 300 − 1400 Da with a resolution of 70,000 at m/z 200. The isolation width was 3 m/z for precursor ion selection. The AGC was set to 3 × 10^6^, and the MIT was 60 ms. The MS2 was analyzed using data-dependent mode searching for the 20 most intense ions fragmented in the HCD. For each scan with a resolution of 17,500 at m/z 200, the AGC was set at 5 × 10^4^ and the MIT was 80 ms. The dynamic exclusion was set at 18 s to suppress repeated detection of the same fragment ion peaks. The relative collision energy for MS2 was at 27% for HCD. The MS data were searched against a Uniprot Mouse database (v20200319) by Mascot software (v2.6.0). The search parameters were as follows: the proteolytic enzyme was trypsin with a maximum of missed cleavages of 3; the peptide charge was set to 2^+^ and 3^+^; the peptide error tolerance was 10 ppm; the MS/MS error tolerance was 0.02 Da; and carbamidomethylation of cysteine (+57.02146 Da) as a fixed modification and acetyl of protein N terminal (+42.010565 Da) and oxidation (M) (+15.994915 Da) were the variable modifications. The peptide and protein FDRs were set to 0.01 by using PepDistiller and PANDA, respectively. The cutoff ion score was set to 10. The identified proteins were scored by SAINTexpress with a cutoff value ≥ 0.9. The network was visualized by Cytoscape (v3.9.1) (40).

### Immunoprecipitation

The cells were harvested and washed with PBS. They were then diluted in NETN lysis buffer (1M Tris–HCl, pH 8.0, 0.5 M EDTA, 5M NaCl, and 0.5% NP-40), supplemented with 1 mM DTT, 1 mM NaF, 2 mM Na_3_VO_4_, and a complete protease inhibitor cocktail. The cells were lysed by sonication, and the lysate was centrifuged at 14,600 rpm for 10 min at 4 °C. Sixty microliters of supernatant were mixed with 60 μl of 2 × loading buffer and heated at 100 °C for 10 min. The remaining supernatants were transferred to new tubes, and protein A/G Magnetic Beads, preincubated with antibody for 2 h, were added and mixed overnight. The magnetic beads were washed with NETN lysis buffer and PBST. The beads were resuspended with NETN lysis buffer, and an equal volume of 2 × loading buffer was added. The samples were boiled at 100 °C for 10 min. Finally, the immunoprecipitated proteins were detected by Western blot.

### RNA Interference

The double-stranded RNA duplexes were synthesized by GenePharma. The small interfering RNA (siRNA) oligonucleotides used to target mouse *Fam120a* were UAA UCA GCU ACG GCU UUG ATT (5′-3′) and UAU ACC GUA AGA CGC AGC ATT (5′-3′). Duplexes of siRNA were transfected into cells using Lipofectamine RNAiMAX Reagent (Invitrogen) according to the manufacturer’s instructions. A nonsilencing duplex was used as a control. Forty-eight hours after transfection, the cells were harvested. BMDMs from 6 to 8-week-old mice were obtained after a 7-day culture with M-CSF (PeproTech). To examine the effect of silencing *Fam120a* on NLRP3 inflammasome activation in BMDM cells, the cells were transfected with 40 μM siRNA. Forty-eight hours later, the cells were primed with LPS for 6 h and activated with nigericin for 30 min. Subsequently, the cells were lysed for Western blot analysis.

### Real-time qPCR

Total RNA from BMDM cells was isolated using TRIzol reagent (Sigma-Aldrich), and complementary DNA was synthesized using the RT Master Mix (TOYOBO). Quantitative PCR was performed using the iQ5 and SYBR Green Detection system (Bio-Rad) on a qTOWER^3^ (Analytik Jena). Each PCR reaction mixture contained 40 ng of complementary DNA template and 10 μM primers in 5 μl of SYBR Green Realtime PCR Master Mix (TOYOBO). Expression values were normalized to those obtained with the control gene *Actb* (encoding beta-actin). The R^2^ (coefficient of determination) for reactions with specific primers was >0.99, and the PCR reaction efficiency ranged from 100% ± 10%, as specified in the Thermo Fisher Scientific protocol. Changes in gene expression levels were calculated using the 2^−ΔΔCt^ method. Data from multiple runs (n ≥ 3) were plotted using GraphPad software.

### Cytotoxicity and Cell Viability Assay

The relevant cells were treated as indicated, and cytotoxicity was assessed using the lactate dehydrogenase (LDH) cytotoxicity assay according to the manufacturer’s instructions. Briefly, 50 μl of each sample supernatant was transferred to a 96-well flat-bottom plate in triplicate wells. Then, 50 μl of CytoTox 96 reagent was added to each well. The plate was incubated at room temperature for 30 min before adding 50 μl/well of stop solution. The absorbance of each well was measured at a wavelength of 492 nm, and cytotoxicity (% LDH release) was calculated according to the manufacturer’s protocol.

### Immunofluorescence Imaging

After plasmid transfection or stimulus treatment, cells were washed with cold PBS and fixed with a 4% paraformaldehyde solution at room temperature for 15 min. They were then permeabilized with 0.2% Triton X-100 in PBS on ice for 10 to 15 min. The cells were blocked with 3% bovine serum albumin in PBST buffer (PBS containing 0.1% Tween 20) at room temperature for 1 h. Specific primary antibodies were then added at a dilution of 1:50-1:200 in PBST buffer and incubated overnight at 4 °C. The cells were washed three times with PBS for 5 min each. Fluorescently conjugated secondary antibodies were applied at a dilution of 1:50-1:200 in PBST buffer and incubated at room temperature for 45 min in the dark, followed by three PBS washes. The stained cells were imaged using an LSM880+ELYRAS.1 Confocal Microscope (Zeiss).

### sgRNA Design, Lentivirus Production, and Transduction

sgRNA sequences were designed using CRISPick (https://portals.broadinstitute.org/gppx/crispick/public). Three oligos containing the target sequences were synthesized and inserted into the digested lentiCRISPRv2 construct. sgRNA sequences are as follows:

sg*Fam120a*-1-F: 5′-CACCGCTGTCCGAGCGCCGTGGTGC-3′

sg*Fam120a*-1-R: 5′-AAACGCACCACGGCGCTCGGACAGC-3′

sg*Fam120a*-2-F:5′-CACCGGGACTGGCCCAAAGTGCCGA-3′

sg*Fam120a*-2-R:5′-AAACTCGGCACTTTGGGCCAGTCCC-3′

sg*Fam120a*-3-F:5′-CACCGACTCAGAGGATACCTTACCG-3′

sg*Fam120a-*3-R:5′-AAACCGGTAAGGTATCCTCTGAGTC-3′

HEK293T cell lines were seeded at 60% density and transfected with pMD2G, psPAX2, and the above lentiCRISPRv2 plasmids using lipofectamine 3000 following the manufacturer’s instructions. After incubation for 2 days, supernatants were collected and concentrated by PEG8000 to obtain concentrated viral particles. Seventy-two hours after virus infection, cells were selected using puromycin.

### Constitution of NLRP3 Inflammasome in HEK293T Cells

HEK293T cells were cultured in 24-well plates at 60% confluence. The cells were cotransfected with the following plasmids: pCMV-Myc-pro-IL-1β (100 ng/well), pCMV-Myc-ASC (20 ng/well), pCMV-Myc-pro-caspase-1 (30 ng/well), pCMV-Myc-NLRP3 (50 ng/well), and either pLV-Neo-Flag-FAM120A or the empty pLV-Neo-Flag vector, with a total of 0.8 μg DNA using the PEI transfection reagent. Cell lysates were collected and analyzed by immunoblotting.

### Experimental Design and Statistical Rationale

**Fig. 1:**
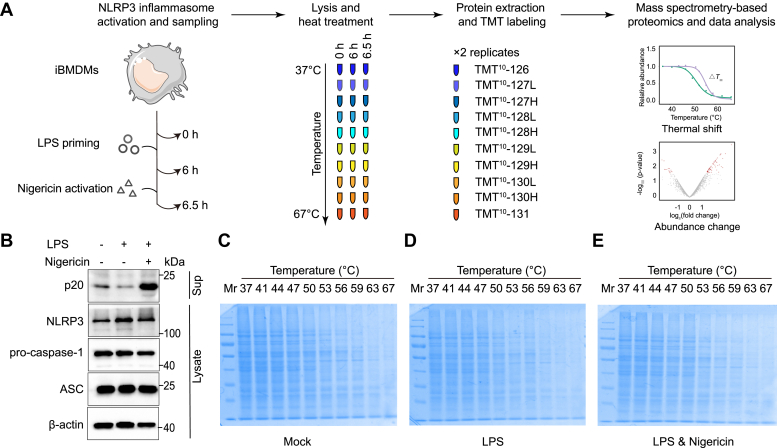
**Global profiling of abundance and thermostability alterations during NLRP3 inflammasome activation**. *A*, schematic overview of the TPP workflow. *B*, immunoblot analysis of iBMDM supernatants and lysates following treatment with LPS and nigericin (Nig). *C*-*E*, the protein levels in supernatants of control (*left*), LPS-primed (*middle*), and nigericin-stimulated cells (*right*) across a temperature gradient by SDS-PAGE. BMDM, bone marrow-derived macrophage; iBMDM, immortalized BMDM; LPS, lipopolysaccharide; NLRP3, NLR family pyrin domain containing; TPP, thermal proteome profiling.

The TPP experimental design is illustrated in Figure 1. We conducted TPP analysis on iBMDM cells under three conditions: untreated, LPS priming for 6 h, and nigericin activation for 0.5 h, each with two biological replicates (n = 2). Following cell lysis, samples were subjected to 10 different temperatures, ranging from 37 °C to 67 °C. A TMT-10 kit was used for labeling one set of biological replicates, with TMT 126 to 131 corresponding to proteome samples treated at each of the 10 temperature points.

For the affinity purification-mass spectrometry (AP-MS) analysis, BMDMs were treated under the same LPS priming and nigericin activation conditions. In the experimental group, NLRP3 antibody was used for enrichment, with three biological replicates (n = 3) for both the LPS-primed and nigericin-activated conditions. In the control group, IgG antibody was used for enrichment, with six biological replicates (n = 6) for each condition. One control replicate was excluded from final data processing due to low correlation, resulting in a final dataset comprising three experimental replicates per condition and eleven control replicates for interaction confidence scoring.

All data are presented as mean ± standard deviation (SD). Statistical comparisons between two groups were conducted using a two-tailed Student’s *t* test, with *p*-values <0.05 considered statistically significant unless otherwise specified. All quantitative experiments were performed in triplicate. Statistical analyses were carried out using GraphPad Prism 9, and detailed statistical information, including sample sizes, is provided in the figure legends. Significance levels are denoted as follows: *p* < 0.05 (∗), *p* < 0.01 (∗∗), *p* < 0.001 (∗∗∗), *p* < 0.0001 (∗∗∗∗).

## Results

### TPP Reveals NLRP3 Inflammasome Activation-dependent Changes in Protein Stability

To investigate the dynamic changes of meltome during NLRP3 inflammasome activation, iBMDM cells were first primed with LPS for 6 h. This duration was chosen to fully activate the Toll-like receptor 4 (TLR4) signaling pathway and ensure sufficient accumulation of key inflammasome components, including NLRP3, pro-IL-1β, and pro-IL-18. Following priming, the cells were stimulated with nigericin for 0.5 h to trigger NLRP3 activation and inflammasome assembly. These time points were selected based on established protocols to capture both the priming and activation phases of inflammasome signaling (Fig. 1*A*). Cleaved caspase-1 (p20) levels in the cell supernatant were assessed *via* Western blot, confirming successful activation of the NLRP3 inflammasome, as evidenced by the expected increasing in p20 level (Fig. 1*B*). Subsequently, the three sample groups, mock, LPS-primed, and nigericin-activated, were subjected to further analysis using the TPP workflow (Fig. 1*A*). To evaluate thermal stability effects on the proteome, cells from each condition were lysed, divided into 10 aliquots, and heated at increasing temperatures ranging from 37 °C to 67 °C. After centrifugation to remove precipitates, supernatants were analyzed by SDS-PAGE (Fig. 1, *C–E*). The results demonstrated a consistent reduction in protein abundance with increasing temperatures across all conditions. Notably, LPS priming and nigericin activation did not significantly alter the overall proteome stability compared to the mock group. The protein samples were then extracted, labeled with 10-plex TMT, and analyzed by quantitative proteomics (Fig. 1*A*). A stringent criterion was used to identify proteins with NLRP3 inflammasome activation-dependent changes in thermal stability. Proteins included in the analysis were required to be identified in at least two biological replicates per condition, which yielded 3628, 3770, and 3640 overlapping proteins for mock, LPS-primed, and nigericin-activated samples, respectively (Supplemental Fig. S1, *A* and *B* and Supplemental Table S1). Consistent with expectations, most proteins exhibited reduced abundance at higher temperatures, with slight differences observed among three groups (Supplemental Fig. S1*C*). The correlation within each group gradually decreased as the temperature increased (Supplemental Fig. S1*D*). A total of 4979 commonly identified proteins across the three sample groups were included for further analysis (Supplemental Fig. S1*E*).

### Comprehensive Proteomic Insights into Thermal Stability and Abundance Changes During NLRP3 Inflammasome Activation

**Fig. 2:**
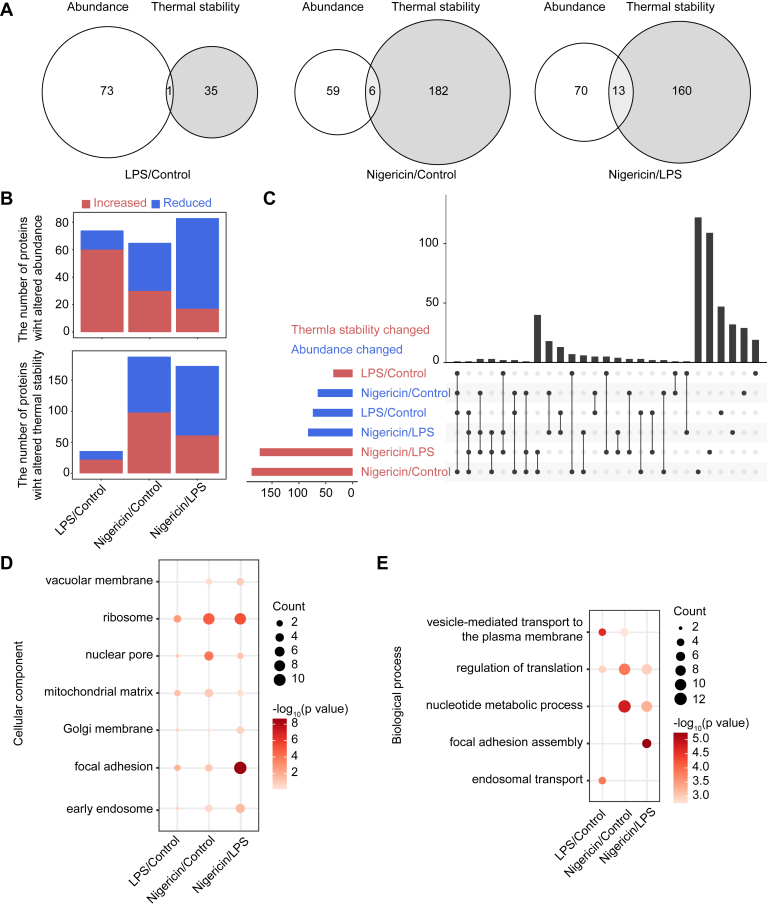
**Characterization of protein abundance and thermostability changes during NLRP3 inflammasome activation**. *A*, Venn diagram illustrating the overlap between proteins with abundance changes and those with altered thermostability in different groups. *B*, bar graph showing trends in protein abundance and thermostability changes in different groups. *C*, UpSet plot summarizing proteins with significant abundance and thermostability changes in different groups. *D*, cellular component (CC) enrichment analysis of proteins with altered thermal stability. *E*, biological process (BP) enrichment analyses of proteins with altered thermal stability. NLRP3, NLR family pyrin domain containing 3

To identify the proteins with thermal stability and abundance changes, the quantitative proteomic data were used to draw thermal shift curves for each protein in mock, LPS-primed, and nigericin-activated samples. The data were analyzed based on Z-scores of the protein and calculated a *T*_m_ value (41). We quantified a total of 3544 proteins with stability Z-scores and 2620 proteins with abundance Z-scores. Proteins with |Z-score| > 1.96 and FDR <0.05 were considered significantly altered. Totally 337 proteins exhibited changes in thermal stability, while 179 showed changes in abundance (Fig. 2*A* and Supplemental Table S2). Compared to mock group or LPS-primed group, the protein abundance or stability of more than half of the differentially proteins are reduced in nigericin-activated group (Fig. 2*B*). Notably, 20 proteins exhibited concurrent changes in both abundance and thermal stability. Compared to mock group, 36 proteins showed changes of thermal stability in LPS-primed group, while 188 proteins exhibited alterations of thermal stability in nigericin-activated group (Fig. 2*A*), suggesting that NLRP3 inflammasome activation induces substantial alterations in protein stability. Conversely, relative to mock group, LPS-primed group exhibited increased abundance and stability for the majority of proteins (Fig. 2*B*). Only minimal overlap was observed between proteins with changes in abundance and thermal stability (Fig. 2*C*), which suggest that thermal stability changes often occur independently of changes in protein abundance.

Thermal stability changes of proteins during NLRP3 inflammasome activation are driven by factors such as protein-protein interactions, posttranslational modifications, and structural alterations, which may play a role in the regulation of the NLRP3 inflammasome pathway. To explore their functions, we analyzed the cellular component and biological process of these 337 proteins with thermal stability changes. We revealed that proteins with stability changes after nigericin treatment were highly enriched in ribosome, Golgi membrane, early endosome and focal adhesion (Fig. 2*D*), and were involved in regulation of translation, nucleotide metabolic process, and focal adhesion assembly (Fig. 2*E*). Considering the essential role of NLRP3 translocation from the endoplasmic reticulum, mitochondria, Golgi apparatus, to focal adhesions, we hypothesized that some proteins exhibiting thermal stability changes directly regulate its activation. Through manually curated literature, we identified 15 proteins with thermal stability changes that have been reported to regulate the activation of NLRP3 inflammasome pathway or interact with its components (Supplemental Table S2*G*). These proteins include the deubquitinases USP9X (42) and USP8 (43), as well as the kinases PTK2B (44), ADK (45), and RIPK3 (46). These results demonstrate that TPP offers an alternative approach for identifying proteins involved in NLRP3 inflammasome activation.

To further verify the reliability of proteins with thermal stability changes during NLRP3 inflammasome activation, we selected three protein with commercially available antibodies and validate them using CETSA. PTK2B (protein-tyrosine kinase 2-beta) and RAF1 (RAF proto-oncogene serine/threonine-protein kinase) showed decreased thermal stability after nigericin treatment, and NUP98 (nuclear pore complex protein NUP98) exhibited increased thermal stability following LPS priming. Mock, LPS-primed, and nigericin-activated cells were lysed, divided into six aliquots, and heated at gradient temperature. The samples were then centrifuged, and the supernatant was analyzed via immunoblotting (Supplemental Fig. S2). Western blot results were quantified and normalized to generate thermal stability curves (Supplemental Fig. S2). Consistent with TPP results, the reducing of thermal stability of PTK2B and RAF1 was confirmed by CETSA (Supplemental Fig. S2, *A* and *B*). The enhanced thermal stability of NUP98 was validated following LPS priming (Supplemental Fig. S2C).

### Hierarchical Clustering Reveals Distinct Protein Thermal Stability Profiles and Functional Pathways During NLRP3 Inflammasome Activation

**Fig. 3:**
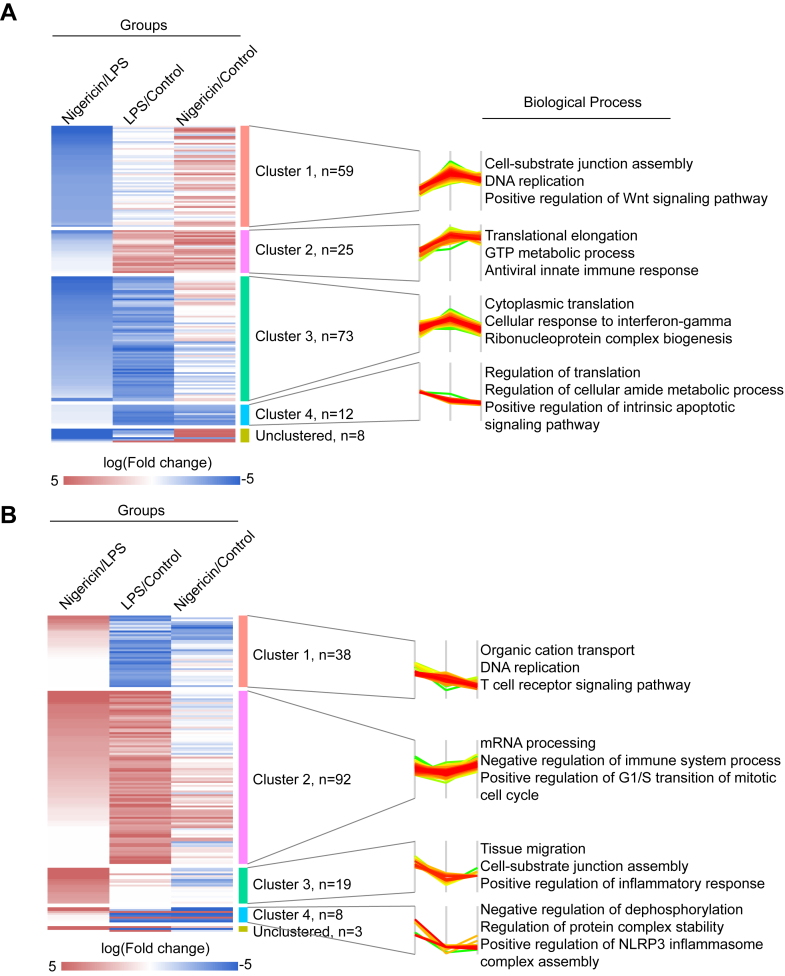
**Thermal stability profiles of proteins during NLRP3 inflammasome activation**. *A*, hierarchical clustering of log fold changes (logFC) for 204 proteins with decreased thermal stability (z-score <0, q-value <0.05). *B*, hierarchical clustering of logFC for 133 proteins with increased thermal stability (z-score ≥0, q-value <0.05). NLRP3, NLR family pyrin domain containing 3.

To further characterize the thermal stability changes of proteins during distinct stages of NLRP3 inflammasome activation, we performed hierarchical clustering of 337 proteins. Compared to the LPS-primed group, these proteins were categorized into two groups based on their thermal stability changes following nigericin treatment: 177 proteins with decreased stability (Fig. 3*A*) and 166 proteins with increased stability (Fig. 3*B*). Based on their dynamic thermal stability patterns during inflammasome activation, these proteins were further classified into five clusters. The representative biological processes enriched in each cluster were shown (Fig. 3). The observed thermal stability shifts in DNA replication, translation, mRNA processing, and cell cycle-related proteins may be attributed to the suppression of these cellular processes under stress conditions. Such suppression allows cells to conserve energy, reallocate resources, and minimize potential damage. Under cellular stress, key biological processes—including transcription, translation, and the cell cycle—are often globally downregulated. For instance, changes in the thermal stability of proteins such as eIF3a and eIF4a2 may indicate their involvement in translation initiation inhibition, while alterations in ribosomal proteins could reflect a broader inhibition of ribosome biogenesis. The differences in protein thermal stability shifts between LPS priming and nigericin activation may reflect distinct cellular adaptive strategies. While LPS primarily activates signal transduction pathways, nigericin treatment induces cell death, leading to differential stabilization or destabilization of key proteins. Among proteins with increased thermal stability after nigericin treatment (Fig. 3*B*), clusters 1, 3, and 4 displayed reduced thermal stability following LPS priming but showed varying degrees of stability enhancement post-nigericin treatment. These proteins participated in immune response-related processes, including T-cell receptor signaling pathway, positive regulation of inflammatory response, and positive regulation of NLRP3 inflammasome complex assembly (Fig. 3*B*). Several proteins in cluster 4 have been previously implicated in NLRP3 inflammasome activation, including MYD88 (47), SEMA4D (48), TBCE (49) and KRT10 (50).

In immune-related processes, specific protein interactions suggest the activation of critical pathways. Notably, MYD88 interacts with CD40, PTK2b, Beclin-1 (BECN1), RIPK3, and CASP7, while TNFRSF10B associates with both RIPK3 and CASP7. These interactions may indicate activation of the NF-κB pathway or inflammasome signaling. Additionally, the transcription factor ZFP36L2, which is induced by NF-κB signaling, undergoes nuclear translocation, where it plays a role in regulating inflammatory gene expression. These results provide insights into the distinct roles and dynamic profiles of proteins involved in thermal stability changes during NLRP3 inflammasome activation.

### Distinct Proteins with Thermostability Changes Are Implicated in Sequential Phases of NLRP3 Inflammasome Activation

**Fig. 4:**
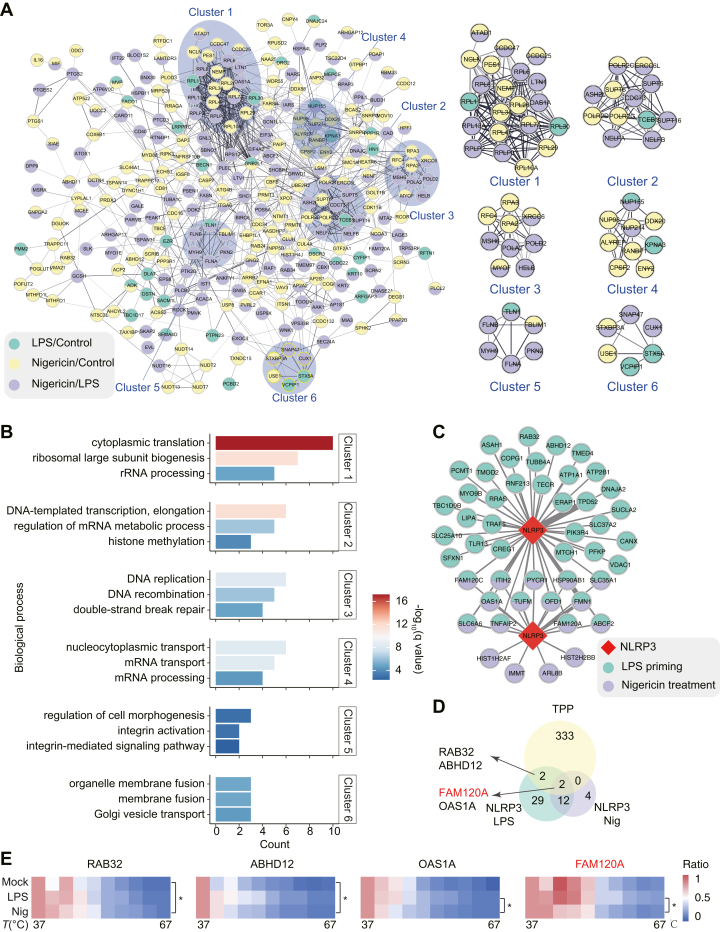
**Network analysis of proteins with thermal stability changes during NLRP3 inflammasome activation**. *A*, a protein-protein interaction network of 286 proteins exhibiting thermal stability changes, constructed using the STRING database. The network highlights six distinct clusters, each representing functionally related proteins. *B*, biological process enrichment analyses for the six identified clusters. Each cluster is associated with specific biological processes, with the *top* three representative processes displayed. *C*, the network of cocomplex proteins associated with NLRP3 proteins through AP-MS. *D*, Venn diagram showing the overlap between TPP and AP-MS datasets. *E*, heatmap of abundance changes of four overlapping proteins identified in (*D*) during NLRP3 inflammasome activation. AP-MS, affinity purification-mass spectrometry; NLRP3, NLR family pyrin domain containing 3; TPP, thermal proteome profiling.

To further explore the functional associations among proteins with thermal stability changes during NLRP3 inflammasome activation, we constructed a protein-protein interaction network using the STRING database (https://www.string-db.org). This analysis identified 967 interactions among 286 proteins (Supplemental Table S3). Applying the Markov Cluster Algorithm within STRING, we delineated six distinct clusters, each associated with specific biological processes (Fig. 4, *A* and *B*). These clusters encompass functions such as translation, DNA replication, mRNA transport, integrin activation, and Golgi vesicle transport. The observed thermal stability changes in proteins related to translation, transcription, and replication (Clusters 1–4) may reflect a downregulation of these processes under cellular stress conditions. Notably, all of the proteins associated with integrin activation (Cluster 5) displayed thermal stability changes after nigericin treatment (Fig. 4*A*). This finding aligns with previous studies demonstrating the role of integrin activation-related proteins in NLRP3 inflammasome activation (51). Similarly, proteins involved in Golgi vesicle transport (Cluster 6) also exhibited thermal stability changes during inflammasome activation. These results support that disruptions in the trans-Golgi network facilitate NLRP3 inflammasome activation (52). These results suggest that specific sets of proteins with thermal stability changes might be functionally associated with distinct stages of NLRP3 inflammasome activation.

It has been demonstrated that proteins within a complex often exhibit comelting behavior, with small molecules influencing the overall stability of these complexes (25). To explore this phenomenon in the context of NLRP3 inflammasome activation, we matched the proteins with thermal stability changes to a manually annotated protein complexes database (CORUM) (53) (Supplemental Fig. S3). Notably, half of the components of the Parvulin-associated pre-rRNP complex exhibited thermal stability changes, which might be due to the stress-induced shutoff of protein translation. Other identified protein complexes contains at least one member related to inflammatory processes, including AKT-ARRB2-PPP2CA-PPP2R2A, RAF1-PP2A core enzyme complex, ARRB1-BECN1-PIK3C3, SNARE complex, SOS1-ABI1-EPS8 complex, and RACK1-containing mRNP complex (Supplemental Fig. S3). These findings suggest that the activation of the NLRP3 inflammasome disrupts the stability of protein complexes, highlighting the potential role of protein complex perturbations in mediating inflammasome activation and its downstream effects.

### Identification of NLRP3 Complex by AP-MS

To find regulators with thermal stability changes during NLRP3 inflammasome activation, we employed AP-MS to identify NLRP3-associated protein complex. BMDM cells were stimulated under the same conditions as TPP, and the proteins were enriched using an NLRP3 antibody and identified by mass spectrometry. We revealed 45 proteins with high-confidence following LPS stimulation, which decreased to 18 after nigericin treatment (Fig. 4*C*, Supplemental Table. S4 and S5)). Four proteins, RAB32, ABHD12, OAS1A, and FAM120A were commonly obtained by AP-MS and TPP (Fig. 4*D*). RAB32 and ABHD12 associated with NLRP3 following LPS stimulation, while OAS1A and FAM120A interacted with NLRP3 under both LPS priming and nigericin treatment conditions (Fig. 4*E*). These findings suggest that these proteins may directly interact with NLRP3 inflammasome and regulate its activation. RAB32 induces unfolded protein response during multiple sclerosis and experimental autoimmune encephalomyelitis, alters mitochondrial morphology, and promotes apoptosis/necroptosis (54). ABHD12 metabolizes lysophosphatidylserine (lyso-PS), and its inhibition by the genetic deletion elevates lyso-PS levels and enhances immune responses (55). OAS1A is linked to type I interferon signaling and may trigger islet autoimmunity and the development of type 1 diabetes (56). FAM120A is reported to protect cells from oxidative stress-induced apoptosis via activation of survival pathways (e.g., Src-family kinases and AKT-mediated pathways (57). Unlike RAB32, ABHD12, and OAS1A, which are already implicated in immune response or inflammatory disease contexts, FAM120A has not been previously associated with immune response, which makes it a compelling target for exploring novel regulatory mechanisms in NLRP3 inflammasome activation.

### FAM120A Interacts with NLRP3 and ASC

**Fig. 5:**
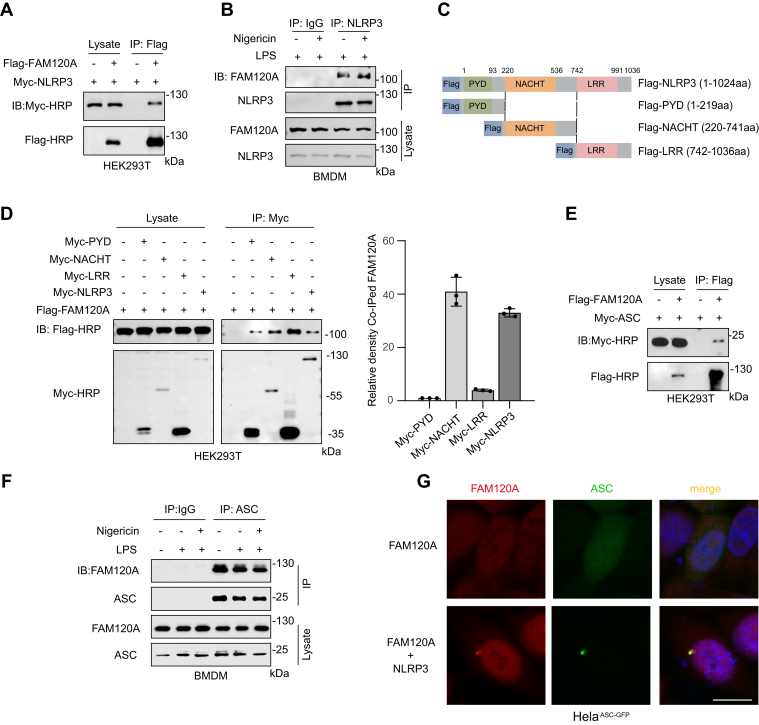
**FAM120A binds to NLRP3**. *A*, coimmunoprecipitation of Myc-tagged NLRP3 with Flag-tagged FAM120A in HEK293T cells, followed by immunoblot analysis. *B*, immunoprecipitation of NLRP3 from BMDMs treated with LPS or nigericin, analyzed by immunoblotting with indicated antibodies. *C*, schematic representation of FLAG-tagged NLRP3 constructs: full-length NLRP3, PYD (amino acids 1–219), NACHT (amino acids 220–741), and LRR (amino acids 742–1036). *D*, coimmunoprecipitation of Flag-tagged FAM120A with Myc-tagged full-length or truncated NLRP3 constructs in HEK293T cells, analyzed by immunoblotting. The coimmunoprecipitation signals were normalized to the relative density of coimmunoprecipitated FAM120A, with quantifications based on triplicate experiments. *E*, interaction of Myc-tagged ASC with Flag-tagged FAM120A analyzed by co-immunoprecipitation in HEK293T cells. *F*, endogenous interaction of ASC and FAM120A analyzed by immunoprecipitation in BMDMs. *G*, confocal imaging of FAM120A (*red*) and ASC-GFP (*green*) in HeLa cells transfected with NLRP3 and/or FAM120A. The scale bar represents 10 μm. ASC, apoptosis-associated speck-like protein containing a CARD; BMDM, bone marrow-derived macrophage; LPS, lipopolysaccharide; LRR, leucine-rich repeat domain.

Given the central roles of NLRP3 and ASC in inflammasome activation, we investigated whether FAM120A interacts with them. We demonstrated that FAM120A directly interacts with NLRP3 by coimmunoprecipitation assays (Fig. 5*A*). The endogenous interaction between FAM120A and NLRP3 was further validated in primary macrophages and detected within NLRP3 protein complexes isolated from BMDMs treated with LPS alone or LPS followed by nigericin stimulation (Fig. 5*B*). To further define the interaction domain, myc-tagged full-length and truncated NLRP3 constructs were generated (Fig. 5*C*). We revealed that FAM120A interacts with all three domains of NLRP3. After normalizing the coimmunoprecipitation signal, we found that NLRP3 primarily interacts with FAM120A through the NACHT domain (Fig. 5*D*). Moreover, we found that FAM120A interacts with ASC (Fig. 5*E*). Notably, NLRP3 interacted with ASC both in the resting state and upon activation by stimulation (Fig. 5*F*). The colocalization of FAM120A with ASC specks suggests its involvement in NLRP3 inflammasome assembly (Fig. 5*G*). Together, these results indicate that FAM120A associates with key proteins of the NLRP3 inflammasome pathway, supporting its potential roles in regulating inflammasome activation.

### FAM120A Inhibits NLRP3 Inflammasome Activation

**Fig. 6:**
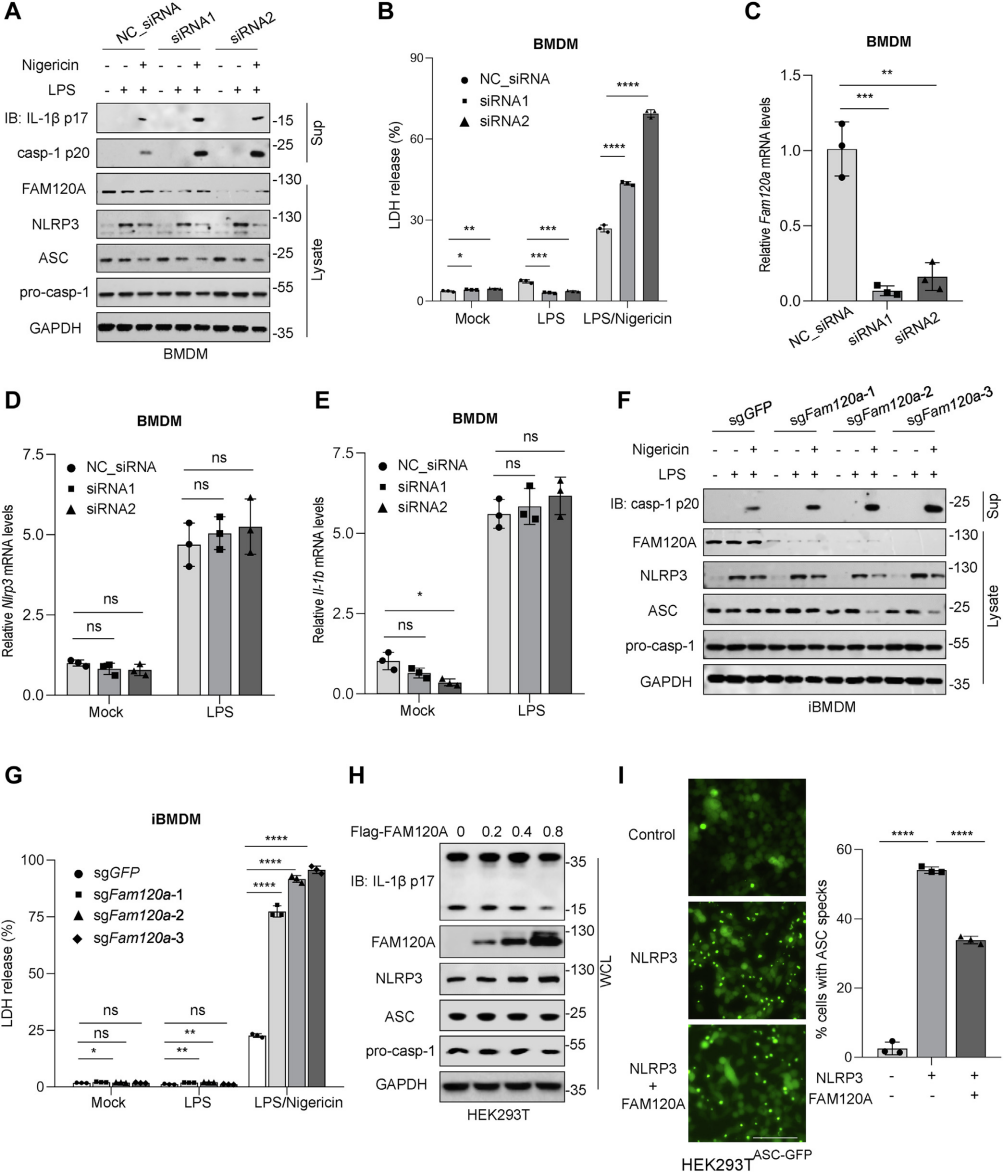
**FAM120A inhibits NLRP3 inflammasome activation**. *A*-*B*, BMDMs treated with control siRNA or *Fam120a* siRNA were stimulated with mock, LPS, or nigericin. Supernatants (Sup) and lysates were analyzed by immunoblotting with indicated antibodies. Cell viability was measured by LDH assay of supernatants. Error bars represent SD of triplicate repeats. Unpaired two-tailed Student’s *t* test: ∗, *p* < 0.05; ∗∗, *p* < 0.01; ∗∗∗, *p* < 0.001; ∗∗∗∗, *p* < 0.0001. *C*-*E*, quantification of relative mRNA levels of *Fam120a* (*C*), *Nlrp3* (*D*), and *Il-1b* (*E*) in *Fam120a* knockdown BMDMs with mock, LPS, or nigericin-treatment. *F*-*G*, NLRP3 inflammasome activation in iBMDMs with control (sg-GFP) or *Fam120a* KO (sg-*Fam120a*). Supernatants and lysates were analyzed by immunoblotting. Cell viability was measured by LDH assay of supernatants. Unpaired two-tailed Student’s *t* test: ∗, *p* < 0.05; ∗∗, *p* < 0.01; ∗∗∗, *p* < 0.001; ∗∗∗∗, *p* < 0.0001. *H*, NLRP3 inflammasome was reconstituted in HEK293T cells by coexpressing of Myc-tagged NLRP3, pro-IL-1b, ASC, pro-caspase-1, and increasing amounts of Flag-tagged FAM120A. The cell lysates were analyzed by immunoblotting in whole-cell lysates (WCL) with indicated antibodies. *I*, ASC-GFP was stably expressed in HEK293T cells. ASC specks were visualized and quantified by fluorescence microscopy of cells transfected with NLRP3 and/or FAM120A. The scale bar represents 100 μm. Error bars represent SD from triplicate replicates. Unpaired two-tailed Student’s *t* test: ∗∗∗∗, *p* < 0.0001. ASC, apoptosis-associated speck-like protein containing a CARD; BMDM, bone marrow-derived macrophage; iBMDM, immortalized BMDM; LDH, lactate dehydrogenase; LPS, lipopolysaccharide; NLRP3, NLR family pyrin domain containing 3; siRNA, small interfering RNA.

To investigate the role of FAM120A in NLRP3 inflammasome activation, we knocked down endogenous *Fam120a* expression by used siRNA in iBMDMs. The cells were stimulated with LPS and nigericin to activate NLRP3 inflammasome pathway. Compared to the control group, we revealed that knockdown of *Fam120a* did not alter the protein levels of NLRP3, ASC, or pro-caspase-1 by Western blot analysis (Fig. 6*A*). However, suppression of *Fam120a* significantly enhanced caspase-1 activation (p20), IL-1β secretion (Fig. 6*A*), and cell death (Fig. 6*B*) following NLRP3 inflammasome activation. Quantitative PCR analysis confirmed effective knockdown of *Fam120a* at the mRNA level (Fig. 6*C*). Importantly, the mRNA levels of *Nlrp3* and *Il-1b* remained unaffected by *Fam120a* knockdown (Fig. 6, *D* and *E*), indicating that FAM120A does not influence the NF-κB-dependent priming phase of inflammasome activation.

To further validate these findings, we generated *Fam120a*-deficient cells using CRISPR/Cas9-mediated gene editing. *Fam120a*-deficient cells exhibited significantly increased caspase-1 cleavage (Fig. 6*F*) and LDH release (Fig. 6*G*), further demonstrating its role as a negative regulator of inflammasome activation. Next, we assessed the role of FAM120A in a cellular model of NLRP3 inflammasome autoactivation. HEK293T cells were cotransfected with NLRP3, ASC, pro-caspase-1, and pro-IL-1β, with NLRP3 inflammasome activation measured by IL-1β cleavage (58). Western blot analysis showed that increasing FAM120A protein levels significantly reduced IL-1β maturation (Fig. 6*H*). We further generated a stable ASC-GFP-expressing HEK293T cell line. Cotransfection with Myc-NLRP3 and Flag-FAM120A revealed that FAM120A reduced the number of ASC specks, an indicator of inflammasome assembly. Quantification results showed 2.58% ASC speck-positive cells in the control group, 54.08% in the Myc-NLRP3 group, and 33.85% in the Myc-NLRP3 and Flag-FAM120A coexpression group (Fig. 6*I*). Collectively, these findings indicate that FAM120A serves as a negative regulator of NLRP3 inflammasome activation by attenuating downstream processes such as caspase-1 activation, IL-1β maturation, and ASC speck formation.

## Discussion

Despite substantial intracellular protein conformational changes and protein-small molecule interactions occurring during NLRP3 inflammasome activation, high-throughput methods to examine the thermodynamic stability of the proteome during this process remain underdeveloped. In this study, we present a streamlined TPP approach to investigate the proteome-wide effects of LPS and nigericin on protein thermostability during NLRP3 inflammasome activation. Our findings map the regulatory proteome landscape underlying NLRP3 inflammasome activation, providing a more comprehensive understanding of its mechanism and implications for NLRP3-associated inflammatory diseases.

Using TPP, we identified 337 proteins with altered thermal stability during inflammasome activation, highlighting the multifaceted regulatory networks involved. Pronounced changes in protein stability were observed across various stages of activation, particularly in endosome, nuclear pore, Golgi membrane, and focal adhesion proteins, consistent with prior findings implicating these cellular structures in NLRP3 activation (59, 60). Of note, 15 proteins among the 337 identified have previously been reported to influence NLRP3 activation, supporting the validity of our approach and offering critical targets for functional exploration. However, we acknowledge that altered protein stability may reflect cellular defense mechanisms rather than direct roles in inflammasome regulation, emphasizing the need for careful evaluation.

Protein complexes are central to cellular function, dynamically assembling and disassembling in response to physiological demands and often implicated in disease states (61, 62). Consistent with previous reports of NLRP3 complex formation during activation (63), our analysis of CORUM complex suggests that perturbations in NLRP3 inflammasome activation influence the thermal stability of associated protein complexes. These findings underscore the intricate interplay between protein stability and complex dynamics during inflammasome activation.

We identified FAM120A as a novel regulator interacting with NLRP3 and ASC. Coimmunoprecipitation and immunofluorescence experiments confirmed FAM120A’s interaction with inflammasome components and colocalization with ASC specks, which demonstrate that FAM120A is involved in the assembly of the NLRP3 inflammasome. Moreover, we proved that FAM120A negatively regulates NLRP3 activation, reducing caspase-1 cleavage, IL-1β secretion, and ASC speck formation. The constitutive interaction between FAM120A and ASC suggests that FAM120A may function as a scaffold in the regulation of the NLRP3 inflammasome rather than being strictly activation-dependent. This interaction remains present under both resting and activated conditions, aligning with our AP-MS data, which also indicate a stable association. These findings suggest that FAM120A may play a regulatory role in inhibiting NLRP3 inflammasome assembly and function. In conclusion, these findings suggest FAM120A as a potential therapeutic target for NLRP3-related inflammatory diseases.

This study reveals physical changes in the proteome during NLRP3 inflammasome activation and highlights FAM120A as a novel negative regulator of inflammasome assembly and function. Our findings underscore the potential of TPP to uncover new insights into the regulation of protein complexes and offer a valuable resource for understanding NLRP3 activation mechanisms.

### Limitations of the Study

The sensitivity of TPP is inherently limited, as some protein interactions or ligand-binding events may not significantly alter thermal stability, and trace changes are challenging to detect. Additionally, while AP-MS complemented TPP by refining the identification of proteins involved in NLRP3 interactions, key components directly implicated in inflammasome assembly remain unidentified. Many identified molecules, including those not previously reported, require further validation and functional studies. Finally, while FAM120A emerges as a promising target, its precise molecular mechanism and clinical applicability need to be further explored. Future research should investigate the broader implications of our findings and the potential for leveraging TPP in diverse biological contexts.

## Data Availability

All proteomics raw data have been deposited to the ProteomeXchange Consortium (https://proteomecentral.proteomexchange.org) via the iProX partner repository (64, 65) with the dataset identifier PXD063269.

## Supplemental Data

This article contains supplemental data.

## Conflict of Interest

The authors declare no competing interests.
