# Supplementary material for: Thermal Proteome Profiling Reveals Meltome Upon NLRP3 Inflammasome Activation
Source: Mol Cell Proteomics. 2025 Apr 16;24(5):100972. doi: 10.1016/j.mcpro.2025.100972 (PMC12148412; doi:10.1016/j.mcpro.2025.100972)
Supplement: Supplemental Figures [file mmc6.pdf]

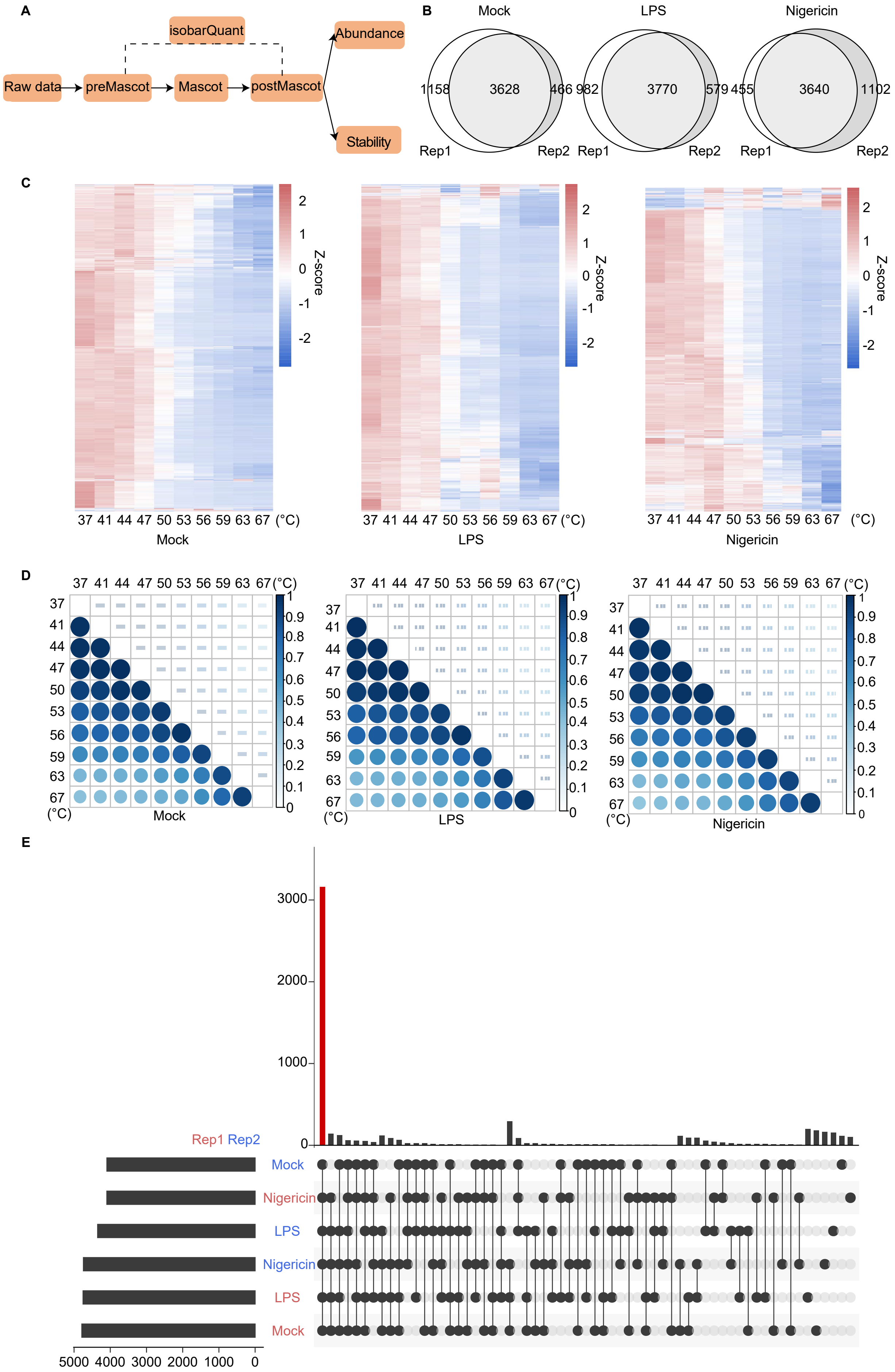

A
isobarQuant
Raw data
preMascot
Mascot
postMascot
Abundance
Stability
B
Mock
LPS
Nigericin
1158
3628
466
982
3770
579
455
3640
1102
Rep1
Rep2
C
Z-score
37 41 44 47 50 53 56 59 63 67 (°C)
Mock
LPS
Nigericin
D
(°C)
E
Rep1 Rep2
Mock
Nigericin
LPS
Nigericin
LPS
Mock
5000 4000 3000 2000 1000 0
3000
2000
1000
0

Figure S1. Thermal proteome profiling of iBMDM cells upon NLRP3 inflammasome activation.
(A) Schematic workflow of the TPP data analysis.
(B) Venn diagram illustrating the overlap of identified proteins across two replicates for each treatment condition.
(C) Heatmap depicting the thermal stability profiles of soluble proteins in control cells (left), LPS-primed cells (middle), and nigericin-activated cells (right).
(D) Correlation map showing the pairwise correlation across ten temperatures under the three treatment conditions.
(E) UpSet plot illustrating the distribution of identified proteins across treatments.

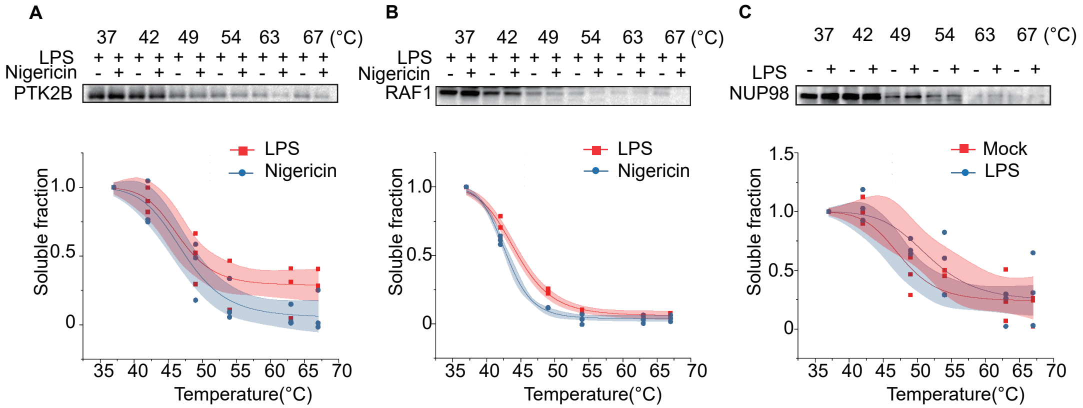

Figure S2. Immunblot analyses and melting curves of three proteins under indicated treatments.

(A) Immunoblot analysis of PTK2B in iBMDM cells treated with LPS or nigericin.

(B) Immunoblot analysis of RAF1 in iBMDM cells treated with LPS or nigericin.

(C) Immunoblot analysis of NUP98 in iBMDM cells treated with mock or LPS.

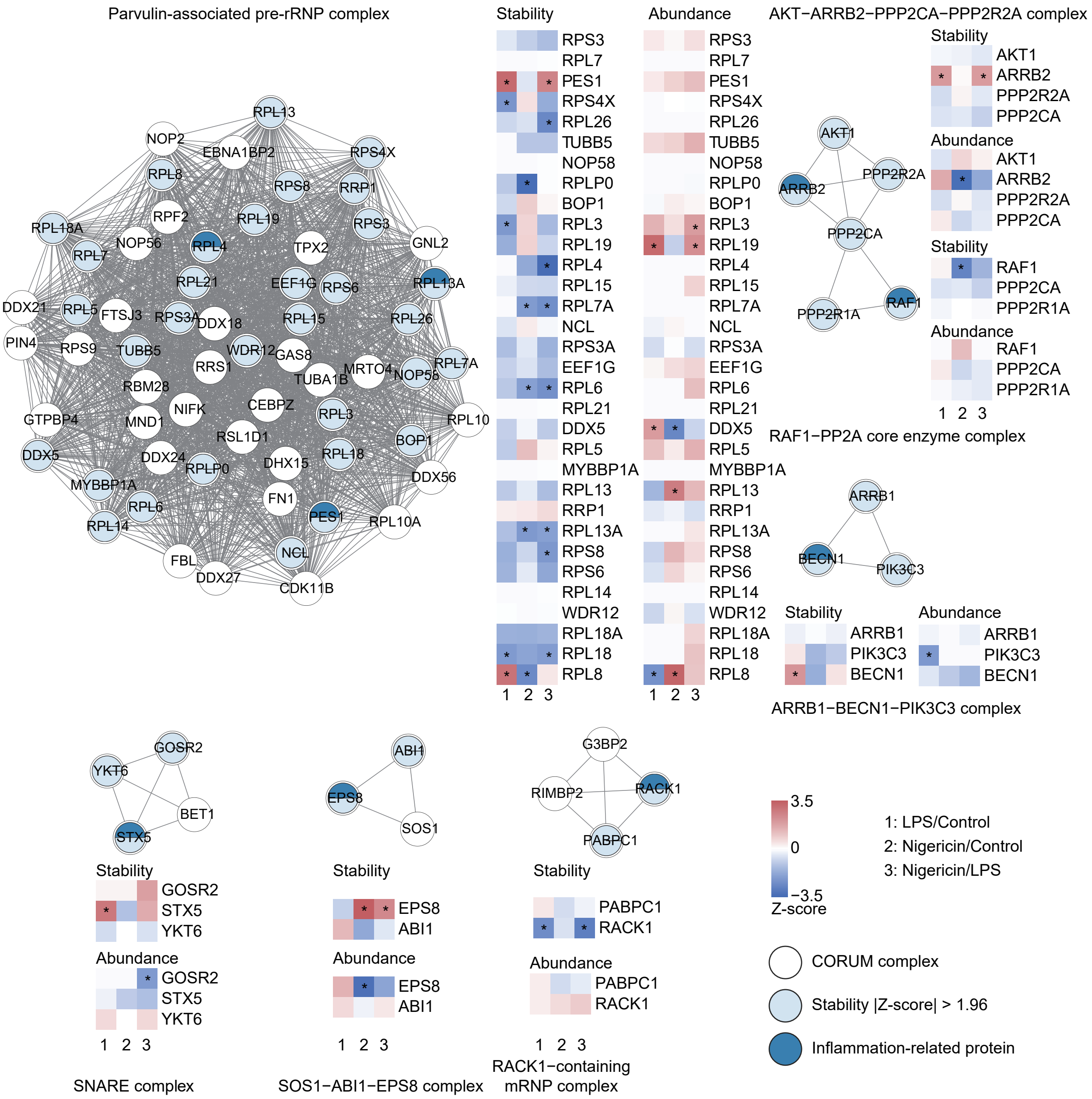

Figure S3. CORUM analysis reveals the protein complexes with altered thermal stability during inflammasome activation. Changes in thermal stability and protein abundance are shown.
